# LYG1 Deficiency Attenuates the Severity of Acute Graft-Versus-Host Disease *via* Skewing Allogeneic T Cells Polarization Towards Treg Cells

**DOI:** 10.3389/fimmu.2021.647894

**Published:** 2021-06-28

**Authors:** Huihui Liu, Zhengyu Yu, Bo Tang, Shengchao Miao, Chenchen Qin, Yuan Li, Zeyin Liang, Yongjin Shi, Yang Zhang, Qingya Wang, Miao Yan, Zhengyang Song, Hanyun Ren, Yujun Dong

**Affiliations:** Department of Hematology, Peking University First Hospital, Beijing, China

**Keywords:** LYG1, aGVHD, allogeneic CD4^+^ T cells, alloreactivity, Th1 cells, Treg cells

## Abstract

Acute graft-versus-host disease (aGVHD) is a lethal complication after allogeneic hematopoietic stem cell transplantation. The mechanism involves the recognition of host antigens by donor-derived T cells which induces augmented response of alloreactive T cells. In this study, we characterized the role of a previously identified novel classical secretory protein with antitumor function-LYG1 (Lysozyme G-like 1), in aGVHD. LYG1 deficiency reduced the activation of CD4^+^ T cells and Th1 ratio, but increased Treg ratio *in vitro* by MLR assay. By using major MHC mismatched aGVHD model, LYG1 deficiency in donor T cells or CD4^+^ T cells attenuated aGVHD severity, inhibited CD4^+^ T cells activation and IFN-γ expression, promoted FoxP3 expression, suppressed CXCL9 and CXCL10 expression, restrained allogeneic CD4^+^ T cells infiltrating in target organs. The function of LYG1 in aGVHD was also confirmed using haploidentical transplant model. Furthermore, administration of recombinant human LYG1 protein intraperitoneally aggravated aGVHD by promoting IFN-γ production and inhibiting FoxP3 expression. The effect of rhLYG1 could partially be abrogated with the absence of IFN-γ. Furthermore, LYG1 deficiency in donor T cells preserved graft-versus-tumor response. In summary, our results indicate LYG1 regulates aGVHD by the alloreactivity of CD4^+^ T cells and the balance of Th1 and Treg differentiation of allogeneic CD4^+^ T cells, targeting LYG1 maybe a novel therapeutic strategy for preventing aGVHD.

## Introduction

Acute graft-*versus*-host disease (aGVHD) is medical complication which mainly destroy host tissues including the skin, liver, colon and the lung after allogeneic hematopoietic cell transplantation (allo-HSCT), representing a major cause for morbidity and non-relapse mortality (1, 2). Alloreactive T cells were the major detrimental factors during the pathogenesis of aGVHD (3, 4). In GVHD, the donor T cells recognize the host antigens, activate, differentiate and traffic to the target organs under guidance of cytokines and chemokines, and result in inflammatory damages in the target organs (5). IFN-γ is a central regulatory cytokine in the initiation and maintenance of aGVHD due to its crucial function for CD4^+^ Th1 cells differentiation and CD8^+^ T cells function during the priming and expansion phase (6). Regulatory T cells (Treg) which reduces the incidence and severity of aGVHD is one of the protective factors against aGVHD (7). Due to the inhibitory characteristics, Treg cells have been widely studied for GVHD treatment in pre-clinical models and clinical trials (8, 9).

Despite considerable achievements in the treatment of aGVHD, it remains a major clinical problem for the patients undergoing allo-HSCT. Approximately 40%-60% of recipients will develop aGVHD, imposing crucial risks for long term survival (10). Because the success of allo-HSCT relies on graft-*versus*-tumor (GVT) function mediated by T cells, immunosuppressive strategies are less attractive (5). Therefore, explorations on new mechanisms and novel therapeutic strategies for aGVHD with preserving GVT responses are important and necessary.

In our previous study, we have identified and characterized a novel classical secretory protein LYG1 (Lysozyme G-like 1) through immunogenomics strategy (11). Recombinant human LYG1 protein (rhLYG1) can inhibit tumor growth by promoting the activation and IFN-γ production of tumor antigen-specific CD4^+^ T cells (11). While LYG1 deficiency accelerated B16 and LLC1 tumor growth due to the inhibited T cell functions. However, the function of LYG1 in other immune diseases is unclear.

Given the enhanced T cell functions under rhLYG1 stimulation and the inhibited T cell functions with LYG1 deficiency, we hypothesized that LYG1 might participate in the development of GVHD. To verify the hypothesis, we explored the role and mechanisms of LYG1 during GVHD using aGVHD murine models in this study.

## Materials and Methods

### Mice

Six- to eight-week-old C57BL/6 (B6, H2Kb) and BALB/c (H2Kd) and (B6 × DBA/2) F1 (BDF1, H-2b–d) mice were purchased from Vital River Laboratories. The *Lyg1* conventional knockout mice (C57BL/6 background, *Lyg1^-/-^*) were generous gifts from Prof. Wenling Han at Peking University Health Science Center (Beijing, China). *IFN-γ^-/-^* mice (B6.129S7-IFNgtm1Ts/J) were purchased from the Model Animal Research Center of Nanjing University. Homozygous knockout (*Lyg1^-/-^*) and the littermate wild-type (WT, *Lyg1^+/+^*) mice were used for all related experiments. All mice were bred at the center animal laboratory of Peking University First Hospital under specific pathogen-free conditions, and all experiments were approved by the Ethics Committee of Peking University First Hospital.

### Mixed Lymphocyte Reaction (MLR)

Splenocytes derived from BALB/c mice were used as stimulator cells. CD3^+^ T cells were used as responder cells selected from splenocytes of *Lyg1^+/+^* or *Lyg1^-/-^* mice using Mouse CD3^+^ T cell isolation kit (Biolegend, San Diego, US) according to the manufacturer’s instructions. The purity of CD3^+^ T cell was >90% assessed by flow cytometry. The responder cells (2 × 10^5^ in 100 μl complete culture medium) labeled by CFSE were cultured with stimulator cells treated with mitomycin C (Selleck, Houston, US) for 30 minutes (5 × 10^5^ in 100 μl complete culture medium) in 96 well plate. After 5 days of culture, the cells were analyzed by flow cytometry.

### aGVHD Mouse Model

Bone marrow cells (BM) were collected by red blood cell lysis. Splenocytes were isolated by Ficoll gradient centrifugation. CD3^+^, CD4^+^ and CD8^+^ T cells were sorted from splenocytes of *Lyg1^+/+^* or *Lyg1^-/-^* mice using Mouse CD3^+^, CD4^+^ and CD8^+^ T cell isolation kit (Biolegend, San Diego, US) according to the manufacturer’s instructions. The purities were >90% assessed by flow cytometry. Recipient (BALB/c, H2Kd) mice were conditioned with total body irradiation (TBI) at 750 cGy (60 Coγ source) on day 0 followed by allogeneic transplantation intravenously: 5 × 10^6^ *Lyg1^+/+^* B6 BM (H2Kb) and 3 × 10^6^ CD3^+^ T cells (or 1.8 × 10^6^ CD4^+^ T cells or 1.8 × 10^6^ CD8^+^ T cells) from *Lyg1^+/+^* or *Lyg1^-/-^* splenocytes (H2Kb) (12). BM control group were given 5 × 10^6^ *Lyg1^+/+^* B6 BM alone. Syngeneic transplant group (Syn) were given 5 × 10^6^ BM (H2Kb) and 3 × 10^6^ CD3^+^ T cells sorting from BALB/c splenocytes (H2Kd). Haploidentical (B6→BDF1) transplant model (Haplo-HSCT): recipient ((B6 × DBA/2) F1 (BDF1, H-2b–d)) mice were conditioned with TBI at 900 cGy on day 0 followed by allogeneic transplantation intravenously: 5 × 10^6^ *Lyg1^+/+^* B6 BM (H2Kb) and 2 × 10^7^ cells from *Lyg1^+/+^* or *Lyg1^-/-^* splenocytes (H2Kb) (13). For the experiments using rhLYG1 administration, recipient (BALB/c, H2Kd) mice were conditioned with TBI at 750 cGy on day 0 followed by allogeneic transplantation intravenously: 5 × 10^6^ B6 BM (H2Kb) and 3 × 10^6^ CD3^+^ B6 or *IFN-γ^-/-^* T cells (H2Kb). rhLYG1 were injected intraperitoneally in BALB/c recipients daily from day 1 to 7 after transplantation. Survival was monitored every day, recipient’s weight and GVHD score were measured every week. The scoring system to evaluate the severity of aGVHD includes five clinical parameters: weight, activity, skin, fur ruffling, and posture. Individual mice were scored 0-2 for each criterion (14). Representative tissues of aGVHD target organs (liver and lung) were excised from recipients on 28 days after transplantation and subjected to histopathological scoring (15, 16). Immunohistochemical (IHC) analysis for CD4+ and CD8+ (servicebio, China) were performed on the tissues of recipient mice at 7 days post-transplantation according to the manufacturer’s instructions.

### GVT Mouse Model

2.5 × 10^4^ mouse mastocytoma cell strain P815 (H2Kd) were injected intravenously into per aGVHD recipient on day 0 after allogeneic transplantation. Survival was monitored every day. The P815 was retrovirally transduced with a *luc/neo* plasmid using a protocol described previously (17). Mice that received P815-*luc/neo* were given intraperitoneal (200 mg/kg) D-Luciferin (Xenogen, Alameda, CA) and placed supine in the Xenogen IVIS bioluminescence imaging system under anesthesia with isofluorane. Pseudocolor images showing whole-body distribution of bioluminescent signal were superimposed on conventional grayscale photographs. Livers were excised from recipients died or 14 days after transplantation and tumor burden on the livers were analyzed.

### Isolation of Cells and Flow Cytometry

Flow cytometry was performed using the following anti-mouse antibodies from Biolegend (Cal., US): H2Kb-FITC, CD3-APC/Cy7, CD4-PE/cy7, CD8-BV421, IFN-γ-PE, CD69-PE, CD44-FITC, CD62L-APC, T-bet-FITC. Spleens, livers and lungs were excised on day 7 after transplantation. Spleens and livers gently pressed through a cell strainer (70 µm). Livers infiltrating lymphocytes were isolated using Percoll (Living, Beijing, China). Perfused lungs were digested in RPMI-1640 medium containing type IV bovine pancreatic DNase (30 mg/ml; Sigma-Aldrich, US) and collagenase XI (0.7 mg/ml; Sigma-Aldrich, US) to obtain single-cell suspensions. Single-cell suspensions prepared from the above operation were kept on ice and blocked by incubation with anti-Fc receptor antibody. For membrane molecule analysis, cells were labeled with fluorescent conjugated antibodies at 4°C for 30 minutes followed by washes with cold PBS. For cytokine analysis, cells were stimulated with Cell Activation Cocktail (with Brefeldin A) (Biolegend, Cal., US) for 6 hours before cells were harvested for analysis. Cells were first stained with surface markers and then fixed and permeabilized with BD IntraSure Kit (BD Biosciences, NJ, US) according to the manufacturer’s instructions for intracellular staining. Foxp3 and T-bet were stained using a Foxp3 Fix/Perm Buffer Set (Biolegend, Cal., US), according to the manufacturer’s instructions. Flow cytometry analysis was performed on FACS Canto II (BD Biosciences, NJ, US) and analyzed with FlowJo software.

### Measurements of Cytokines in Serum

The peripheral blood samples were obtained on day 7 after transplantation and clotted for 5 h at room temperature before centrifugation for 15 minutes at 2000g. The serums were collected and stored at -80°C. The serum concentrations of IFN-γ, TNF-α and IL-6 were quantitated using a mouse Th cytometric bead array kit (BD Biosciences, NJ, US) (Biolegend, Cal., US) according to the manufacturer’s instructions.

### Real-Time Quantitative PCR (qPCR)

qPCR was performed for quantitative analyses in an ABI Prism 7000 Sequence Detection System (Applied Biosystems). Amplifications were performed using Power SYBR™ Green PCR Master Mix (Thermo Fisher Scientific, MA, US). The quantification data were analyzed with ABI Prism 7000 SDS software. The expression levels of the target genes were normalized to the internal standard gene GAPDH using the comparative Ct method (ddCt). Primers used in qPCR to examine the genes:

Foxp3:
Forward Primer (5’–3’) TTTCACCTATGCCACCCTTATC
Reverse primer (5’–3’) CATGCGAGTAAACCAATGGTAG
CCL5
Forward Primer (5’–3’) GTATTTCTACACCAGCAGCAAG
Reverse primer (5’–3’) TCTTGAACCCACTTCTTCTCTG
CXCL9
Forward Primer (5’–3’) AATCCCTCAAAGACCTCAAACA
Reverse primer (5’–3’) TCCCATTCTTTCATCAGCTTCT
CXCL10
Forward Primer (5’–3’) CAACTGCATCCATATCGATGAC
Reverse primer (5’–3’) GATTCCGGATTCAGACATCTCT
GAPDH
Forward Primer (5’–3’) CACCAACTGCTTAGCCCCC
Reverse primer (5’–3’) TCTTCTGGGTGGCAGTGATG

### Statistical Analysis

Survival curve was analyzed using Kaplan-Meier method. Differences between groups in survival studies were determined using log-rank test. A student t test was applied for the other studies. Independent experiment was performed 3 times. The results in the repeats were similar in this study. p < 0.05 is considered statistically significant. *p < 0.05, **p < 0.01, and ***p < 0.001. Analyses were performed using GraphPad Prism 7.

## Results

### LYG1 Deficiency Inhibited Alloreactivity of CD4^+^ T Cells *In Vitro*

**Figure 1:**
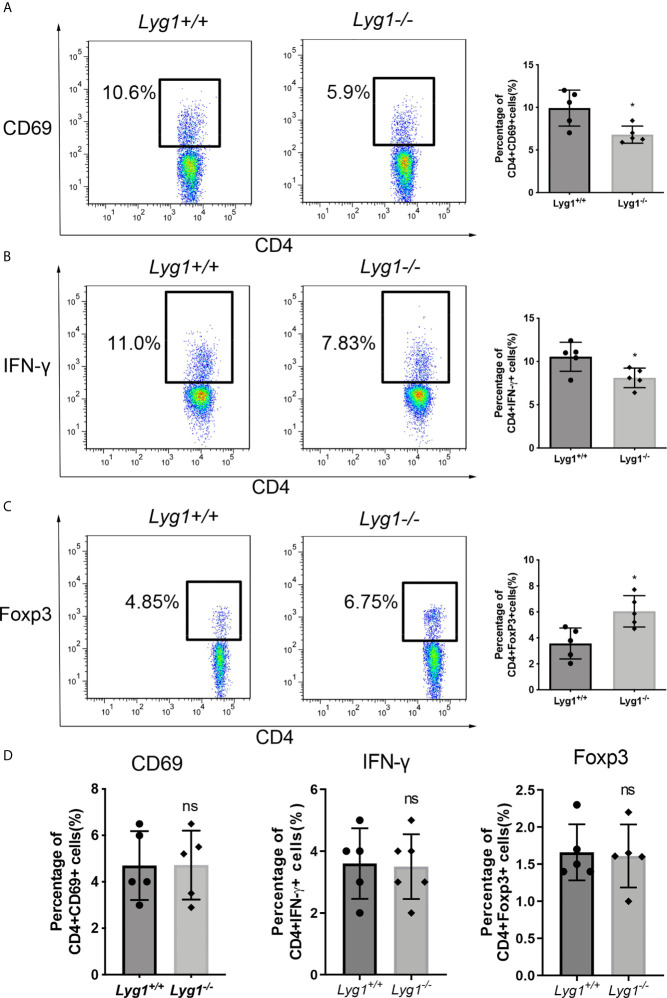
LYG1 deficiency inhibited alloreactivity of CD4^+^ T cells *in vitro*. In MLR assay, CD3^+^ T cells from *Lyg1^+/+^* or *Lyg1^-/-^* mice as responder cells were cultured with mitomycin treated splenocytes from BALB/c mice as stimulator cells. After 5 days, CD69 expression **(A)**, Th1 **(B)** and Treg **(C)** in the responder CD4^+^ T cells were detected by flow cytometry analysis. **(D)** The expression of CD69, IFN-γ and Foxp3 gated on *Lyg1^+/+^* and *Lyg1^-/-^* CD4^+^ T cells prior to the culture. Independent experiment was performed 3 times. n = 5 per group. Representative plots gated on H2Kb+CD4+ T cells are shown and statistical results are expressed as the mean ± SD, *p < 0.05 compared with *Lyg1^+/+^* group. ns, no significance.

Firstly, we examined whether LYG1 affected the alloreactivity of CD4^+^ T cells *in vitro* by MLR assay. The expression of the activation maker CD69 on CD4^+^ T cells were decreased in *Lyg1^-/-^* mice compared with the *Lyg1^+/+^* mice (**Figure 1A**), so was for the IFN-γ production (**Figure 1B**). While the percentages of Treg cells gated on CD4^+^ T cells were higher in *Lyg1^-/-^* group than *Lyg1^+/+^* group (**Figure 1C**). The control group (without stimulating cells) had not response (**Figure 1S**). Whereas there were no differences in the expression of CD69, IFN-γ and Foxp3 between *Lyg1^+/+^* and *Lyg1^-/-^* mice prior to the culture (**Figure 1D**). These results suggest that LYG1 deficiency restrains the alloreactivity of CD4^+^ T cells *in vitro*.

### LYG1 Deficiency in Donor T Cells Alleviated aGVHD

**Figure 2:**
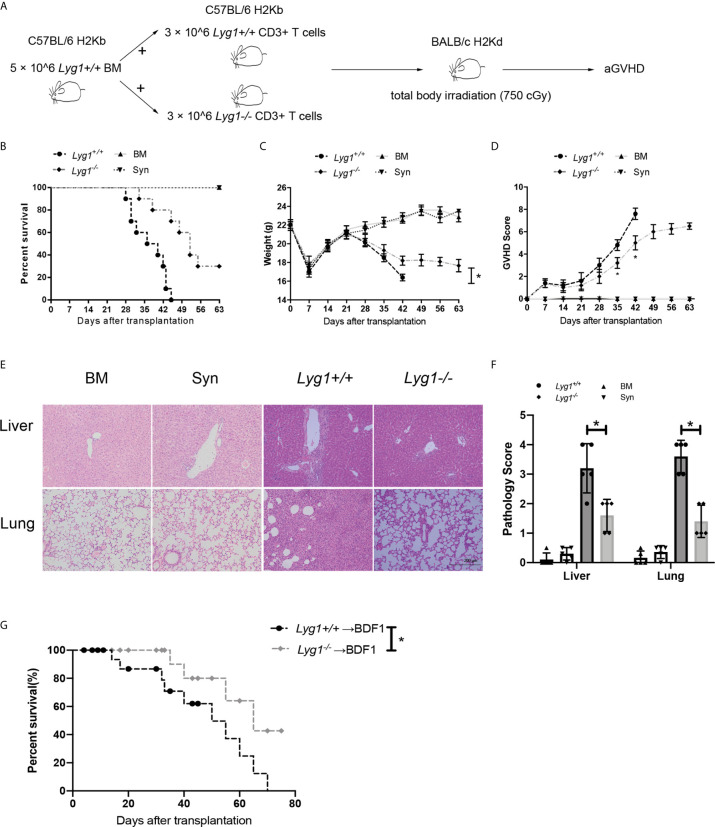
LYG1 deficiency in donor T cells alleviated aGVHD. Lethally irradiated BALB/c mice were reconstituted with 5 × 10^6^ *Lyg1^+/+^* BM and 3 × 10^6^ T cells from *Lyg1^+/+^* mice (*Lyg1^+/+^* group) or *Lyg1^-/-^* mice (*Lyg1^-/-^* group). BM control (BM group) were given 5 × 10^6^ *Lyg1^+/+^* BM alone. **(A)** The diagram illustrating the experimental procedure. Survival **(B)**, weight **(C)** and aGVHD scores **(D)** were monitored. **(E)** Histological examination (×200 magnification) of liver and lung in four groups were analyzed on day 28 after transplantation. **(F)** Histologic scores of liver and lung were shown. **(G)** Survival of BDF1 recipients given transplants with 5 × 10^6^ *Lyg1^+/+^* BM and 2 × 10^7^ splenocytes from *Lyg1^+/+^* mice (*Lyg1^+/+^* group) or *Lyg1^-/-^* mice (*Lyg1^-/-^* group). Independent experiment was performed 3 times. Data pooled: 3 experiments (n = 10 for *Lyg1^+/+^* group and *Lyg1^-/-^* group, n = 5 for BM group). Results are expressed as the mean ± SD, *p < 0.05 compared with *Lyg1^+/+^* group.

We adopted a major MHC mismatched aGVHD model to examine the role of LYG1 in the development of aGVHD (**Figure 2A**). There were no differences in distribution of T cells subsets, including naive (the most dominant subset), central memory and effector CD4^+^ T cells and CD8^+^ T cells, from Lyg1^+/+^ and Lyg1^-/-^ mice before adoptive transfer (**Figure S2**). As shown in **Figures 2B, C**, the control mice in BM group (only transplantation of BM) and Syn group did not induce aGVHD. Comparing with recipients receiving Lyg1^+/+^ T cells, recipients receiving Lyg1^-/-^ T cells showed significantly higher long-term survival rates (**Figure 2B**), less weight loss (**Figure 2C**), and lower aGVHD clinical scores (**Figure 2D**) after 3 weeks since the allogeneic transplantation. Pathology revealed that mice receiving Lyg1^-/-^ T cells showed dramatically reduced inflammation in the livers and lungs compared with those receiving Lyg1^+/+^ T cells (**Figure 2E**). The histological grades of livers and lungs were significantly decreased in recipients receiving Lyg1^-/-^ donor T cells (**Figure 2F**). There was no pathological lesion and inflammation in BM group and Syn group (**Figures 2E, F**). We also examined the LYG1 effect on aGVHD using haplo-HSCT model. The mice receiving Lyg1^-/-^ T cells also exhibited a higher survival rate than did the control mice (**Figure 2G**). These results proved that LYG1 deficiency in donor T cells decreased aGVHD mortality and severity.

### LYG1 Deficiency in Donor T Cells Dampened the Function of Allogeneic CD4^+^ T Cells in Spleens

**Figure 3:**
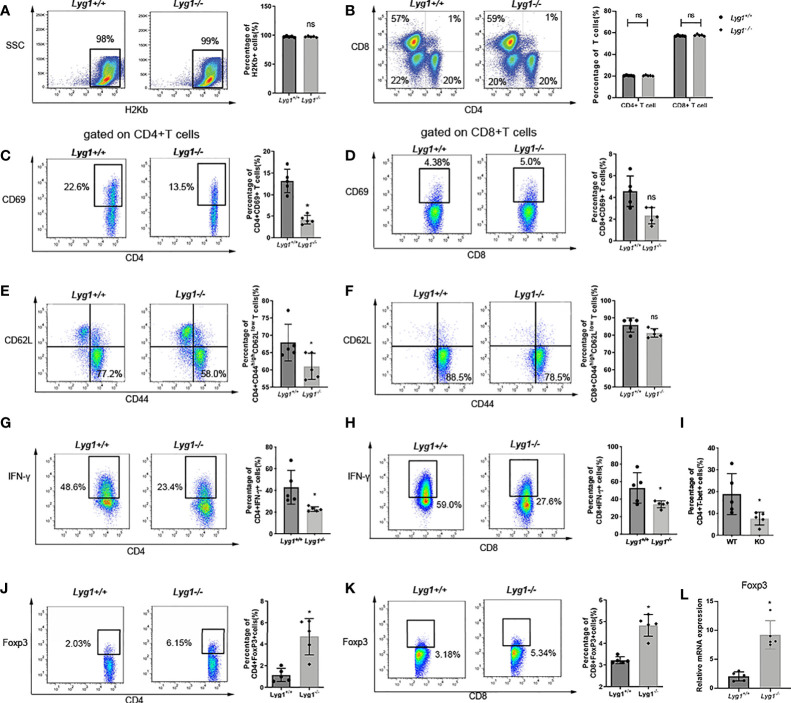
LYG1 deficiency reduced allogeneic T cells function in spleens. Splenocytes of recipient mice were isolated on day 7 after transplantation and analyzed by flow cytometry and qPCR. **(A)** The percentages of H2Kb+ cells in living splenocytes. **(B)** The percentages of CD4^+^ T and CD8^+^ T cells in H2Kb+ splenocytes. **(C, D)** The percentages of CD69 expression in CD4^+^ T cells and CD8^+^ T cells. **(E, F)** The expression of effector (CD44^hi^CD62L^lo^) phenotype gated on CD4^+^ T and CD8^+^ T cells. **(G, H)** The percentages of IFN-γ expression in CD4^+^ T cells and CD8^+^ T cells. **(I)** The percentages of T-bet expression in CD4^+^ T cells. **(J, K)** The percentages of Treg in CD4^+^ T cells and CD8^+^ T cells. The percentages of **Figure 3** **(C–K)** were all gated on H2Kb^+^CD4^+^ cells or H2Kb^+^CD8^+^ cells. **(L)** Foxp3 expression of splenocytes were examined by qPCR. Independent experiment was performed 3 times. The results in the repeats were similar. n = 5 per group. Representative plots are shown and statistical results are expressed as the mean ± SD, *p < 0.05 compared with *Lyg1^+/+^* group. ns, no significance.

First, we examined the donor chimerism in the spleen of recipient mice on day 7 after transplantation. Nearly 98% of H2Kb+ donor cells can be observed in recipient spleens in *Lyg1^+/+^* and *Lyg1^-/-^* aGVHD groups (**Figure 3A**), suggesting LYG1 deficiency in donor T cells did not affect the engraftment of donor cells. To explore potential regulation mechanisms for LYG1 in aGVHD, we investigated the activation and differentiation of H2Kb+ donor T cells from recipients receiving either *Lyg1^+/+^* or *Lyg1^-/-^* T cells. We did not observe notable difference in the absolute number (data not shown) and ratio of CD4^+^ or CD8^+^ T cells between the two groups (**Figure 3B**); however, we noticed significant decrease of CD69 expression on CD4^+^ T in mice receiving *Lyg1^-/-^* T cells (**Figures 3C, D**), suggesting reduced activation of CD4^+^ T cells in these mice. The effector T cells (CD44^hi^CD62L^lo^) also decreased in mice receiving *Lyg1^-/-^* T cells (**Figures 3E, F**). The IFN-γ producing CD4^+^ (Th1) and IFN-γ producing CD8^+^ T cells (Tc1, T cytotoxic 1) were significantly reduced in recipients of *Lyg1^-/-^* T cells compared with those of *Lyg1^+/+^* group (**Figures 3G, H**). T-bet is a master regulator for Th1 differentiation and IFN-γ production (18).Then we examined T-bet and found the percentages of T-bet on CD4^+^ T cells were lower in mice receiving *Lyg1^-/-^* T cells (**Figure 3I**).

Treg cells have been shown to be capable of reducing the severity of aGVHD by restraining immoderate immune activation and maintaining immune homeostasis (19). We found that the proportions of Treg cells (Foxp3+ gated on CD4^+^ T cells) in spleens were dramatically enhanced from recipients received *Lyg1^-/-^* donor T cells (**Figure 3J**), suggesting LYG1 deficiency in donor T cells promotes Treg differentiation. Interestingly, the proportions of Foxp3+ population gated on CD8^+^ T cells increased in spleens in *Lyg1^-/-^* group than *Lyg1^+/+^* group (**Figure 3K**). The mRNA expression of Foxp3 in spleens were also higher in recipients received *Lyg1^-/-^* donor T cells (**Figure 3L**).

We found the similar results in the haplo-HSCT model, LYG1 deficiency in donor T cells decreased the expression of CD69 and IFN-γ, but increased the expression of FoxP3 on T cells (**Figure S3**). Whereas the BM and Syn control group had a lower T cells response (**Figure S4**).

### LYG1 Deficiency in Donor T Cells Inhibited Allogeneic CD4^+^ T Cells Infiltration in aGVHD Target Organs

**Figure 4:**
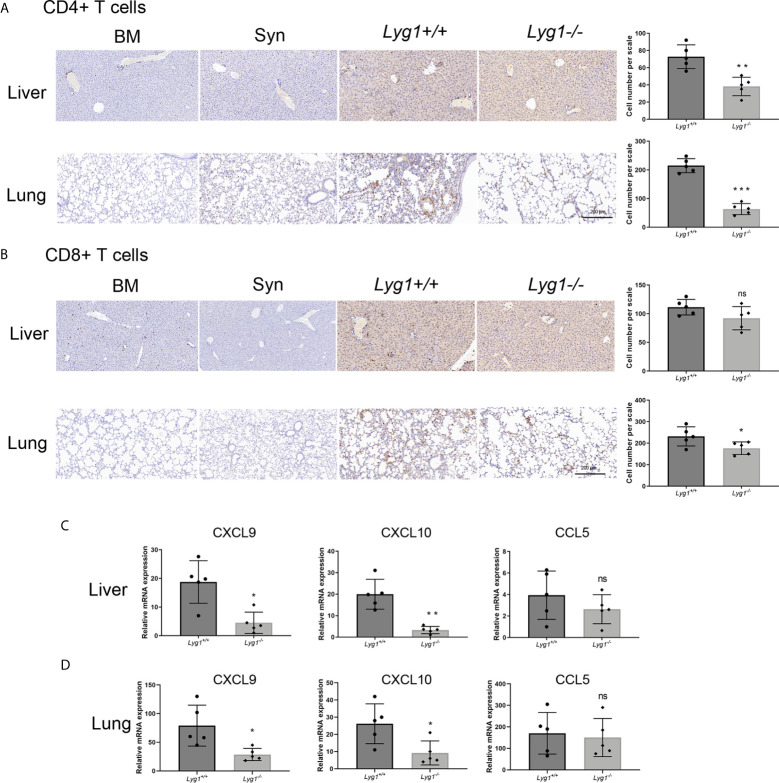
LYG1 deficiency inhibited allogeneic CD4^+^ T cells infiltration in aGVHD target organs. The samples of livers and lungs were excised at day 7 after transplantation and stained with antibodies CD4 and CD8. **(A, B)** The infiltration of CD4^+^ T cells and CD8^+^ T cells in livers and lungs in BM, Syn group, or mice receiving *Lyg1^+/+^* and *Lyg1^-/-^* donor T cells determined by IHC assay (×200 magnification). The left is one representative section per group. The right is the number of CD4^+^ T cells and CD8^+^ T cells per scale in the livers and lungs. **(C, D)** Chemokines expression were examined by qPCR in lymphocytes isolated from livers and lungs. Independent experiment was performed 3 times. The results in the repeats were similar. n = 5 per group. Representative sections are shown and statistical results are expressed as the mean ± SD, *p < 0.05, **P < 0.01 and ***P < 0.001 compared with *Lyg1^+/+^* group. ns, no significance.

We also evaluated the lymphocytes in livers and lungs, the representative target organs of aGVHD. Similarly with spleens, the infiltrating lymphocytes were almost H2kb+ donor cells (data not shown). A significant reduction of CD4^+^ T cells in livers and lungs were observed in mice receiving *Lyg1^-/-^* donor T cells compared with the *Lyg1^+/+^* group determined by IHC (**Figure 4A**). CD8^+^ T cells infiltration in aGVHD target organs also reduced slightly in *Lyg1^-/-^* group (**Figure 4B**). The T cells infiltration in livers and lungs were rarely detected in BM and Syn control group. The decrease of T cells infiltrating in livers and lungs suggested that LYG1 deficiency might change the expression of chemokines that recruited T cells. Therefore, we examined the expression of CCL5, CXCL9, CXCL10 in livers and lungs. LYG1 deficiency inhibited significantly the mRNA expression of CXCL9 and CXCL10, but had no obvious effect on CCL5 expression (**Figures 4C, D**).

### LYG1 Deficiency in Donor T Cells Inhibited the Function of Allogeneic CD4^+^ T Cells in GVHD Target Organs

**Figure 5:**
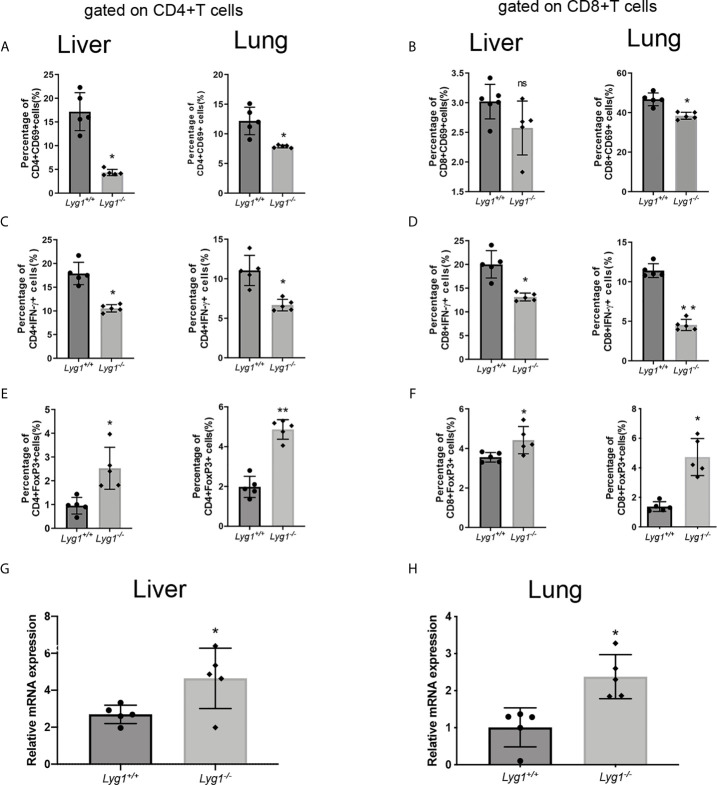
LYG1 deficiency inhibited allogeneic T cells function in livers and lungs. Lymphocytes were isolated from livers and lungs of recipient mice on day 7 after transplantation and analyzed by flow cytometry and qPCR. **(A, B)** The percentages of CD69 expression in CD4^+^ T cells and CD8^+^ T cells. **(C, D)** The percentages of IFN-γ expression in CD4^+^ T cells and CD8^+^ T cells. **(E, F)** The percentages of Treg in CD4^+^ T cells and CD8^+^ T cells. The percentages of **Figure 5** **(A–F)** were all gated on H2Kb+CD4+ cells or H2Kb+CD8+ cells in lymphocytes isolated from livers and lungs. **(G, H)** Foxp3 expression of lymphocytes isolated from livers and lungs were examined by qPCR. Independent experiment was performed 3 times. The results in the repeats were similar. n=5 per group. Statistical results are expressed as the mean ± SD, *p < 0.05 and **P < 0.01 compared with *Lyg1^+/+^* group. ns, no significance.

LYG1 deficiency reduced the number of T cells infiltrating in GVHD target organs, whether it affect allogeneic T cells function? Therefore, we investigated the activation and differentiation of donor T cells in livers and lungs from recipients receiving either *Lyg1^+/+^* or *Lyg1^-/-^* T cells. Similarly, the CD69 and IFN-γ expression of CD4^+^ T cells and CD8^+^ T cells decreased in *Lyg1^-/-^* recipient livers and lungs compared with the *Lyg1^+/+^* groups (**Figures 5A–D**). The percentages of Foxp3+ population gated on CD4^+^ T cells and CD8^+^ T cells were higher in livers and lungs in *Lyg1^-/-^* group than *Lyg1^+/+^* group (**Figures 5E, F**). The expression of Foxp3 in mRNA level in livers and lungs also increased in recipients received *Lyg1^-/-^* donor T cells (**Figures 5G, H**).

### LYG1 Mediated GVHD Development Mainly Through CD4^+^ T Cells

**Figure 6:**
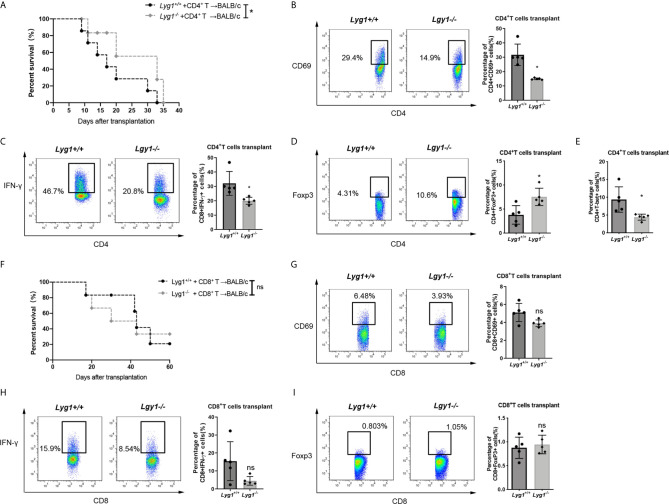
LYG1 mediated aGVHD development mainly through CD4+ T cells. Survival of mice receiving CD4^+^ **(A)** and CD8^+^ **(F)** donor T cells were monitored. Data pooled: n = 10 for *Lyg1^+/+^* group and *Lyg1^-/-^* group **(A, F)**. **(B–E)** The expression of CD69, IFN-γ, Foxp3 and T-bet gated on CD4^+^ T cells. **(G–I)** The expression of CD69, IFN-γ and Foxp3 gated on CD8^+^ T cells. n = 5 per group **(B–E, G–I)**. Representative plots are shown and statistical results are expressed as the mean ± SD, *p < 0.05 compared with *Lyg1^+/+^* group. ns, no significance.

To test whether the effects of LYG1 on GVHD mediated through CD4^+^ T cells or CD8^+^ T cells, we performed GVHD models using purified CD4^+^ T cells or CD8^+^ T cells as grafts, respectively. As illustrated in **Figures 6A, F**, the reduction of aGVHD lethality by LYG1 deficiency was observed in CD4^+^ T cells transplant, but not CD8^+^ T cells transplant. LYG1 deficiency in CD4+ T cells transplant significantly reduced the activation of CD4^+^ T cells and IFN-γ and T-bet expression, but increased Treg ratio (**Figures 6B–E**), but not in CD8^+^ T cells transplant (**Figures 6G–I**). Taken together, the results suggested that LYG1-mediated GVHD development mainly depended on CD4^+^ T cells, but not CD8^+^ T cells.

### rhLYG1 Aggravated aGVHD *via* Promoting IFN-γ Production and Inhibiting Foxp3 Expression

**Figure 7:**
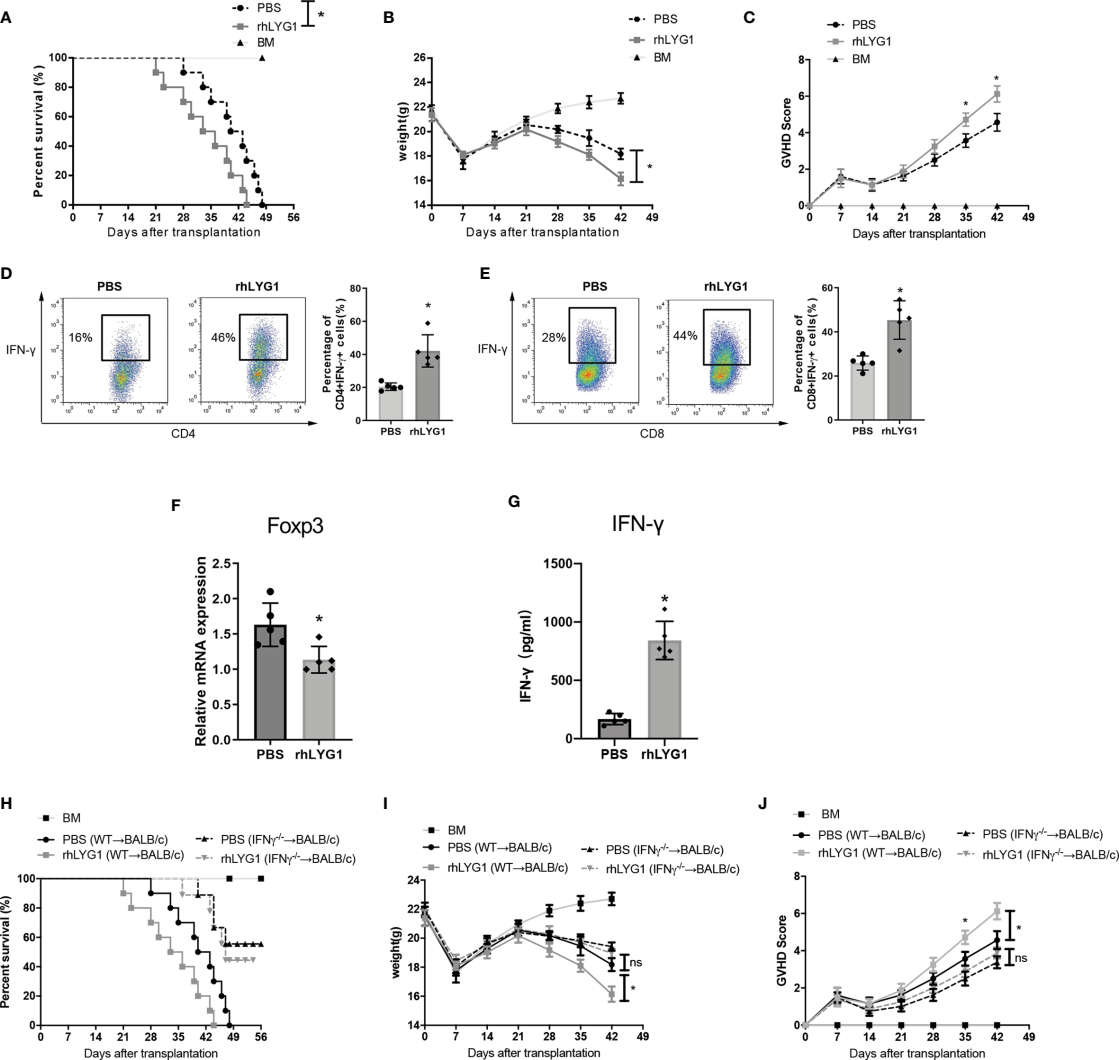
rhLYG1 aggravates aGVHD *via* promoting IFN-γ production and inhibiting Foxp3 expression. **(A–C)** Lethally irradiated BALB/c mice were reconstituted with 5 × 10^6^ B6 BM and 3 × 10^6^ B6 CD3^+^ T cells, rhLYG1 (20 μg per mice) or PBS was injected intraperitoneally (i.p.) each day on days 0 to +7 after transplantation. Survival **(A)**, weight **(B)** and aGVHD scores **(C)** were monitored. **(D, E)** The percentages of IFN-γ positive gated on H2Kb+CD4+ T cells and H2Kb+CD8+ T cells in spleens at day 7 after transplantation. **(F)** Foxp3 expression of splenocytes were examined by qPCR at day 7 after transplantation. **(G)** The concentrations of IFN-γ in serum at day 7 after transplantation. **(H–J)** Lethally irradiated BALB/c mice were reconstituted with 5 × 10^6^ B6 BM and 3 × 10^6^ *IFN-γ ^-/-^* CD3^+^ T cells, rhLYG1 (20 μg per mice) or PBS was injected intraperitoneally (i.p.) each day on days 0 to +7 after transplantation. Survival **(H)**, weight **(I)** and aGVHD scores **(J)** were monitored. **(A–C, H–J)** Data pooled: n = 10 for PBS group and rhLYG1 group, n = 5 for BM group. **(D–G)** Independent experiment was performed 3 times. The results in the repeats were similar. n = 5 per group. Representative plots are shown and statistical results are expressed as the mean ± SD, *p < 0.05 compared with PBS group. ns, no significance.

Furthermore, we used the purified rhLYG1 to evaluate the role of LYG1 in aGVHD model. As shown in **Figures 7A, B**, rhLYG1 significantly accelerated and exacerbated the death and weight loss compared with PBS control. Higher clinical aGVHD scores were seen in rhLYG1 group than in control group (**Figure 7C**). The IFN-γ production of CD4^+^ T cells and CD8^+^ T cells (Th1 and Tc1 cells) were significantly higher than PBS control in spleens (**Figures 7D, E**). The mRNA expression of FoxP3 in spleens decreased in mice treated with rhLYG1 compared with PBS (**Figure 7F**). The IFN-γ concentrations in serum from mice treated with rhLYG1 were higher than that from PBS group (**Figure 7G**). We also detected the IFN-γ, TNF-α and IL-6 in serum in the above GVHD models and found that the concentrations of IFN-γ, TNF-α and IL-6 reduced in recipients received *Lyg1^-/-^* donor T cells compared with recipients received *Lyg1^-/-^* donor T cells, especially for IFN-γ (**Figure S5**). Further we verified the role of IFN-γ in the effects of LYG1 on GVHD using *IFN-γ^-/-^* T cells as grafts. As showed in **Figures 7H–J**, with the deficiency of IFN-γ, the effect of rhLYG1 aggravating aGVHD was partially abrogated, which reconfirmed the crucial role of IFN-γ in LYG1-mediated GVHD development.

### LYG1 Deficiency in Donor T Cells Preserved GVT Response

**Figure 8:**
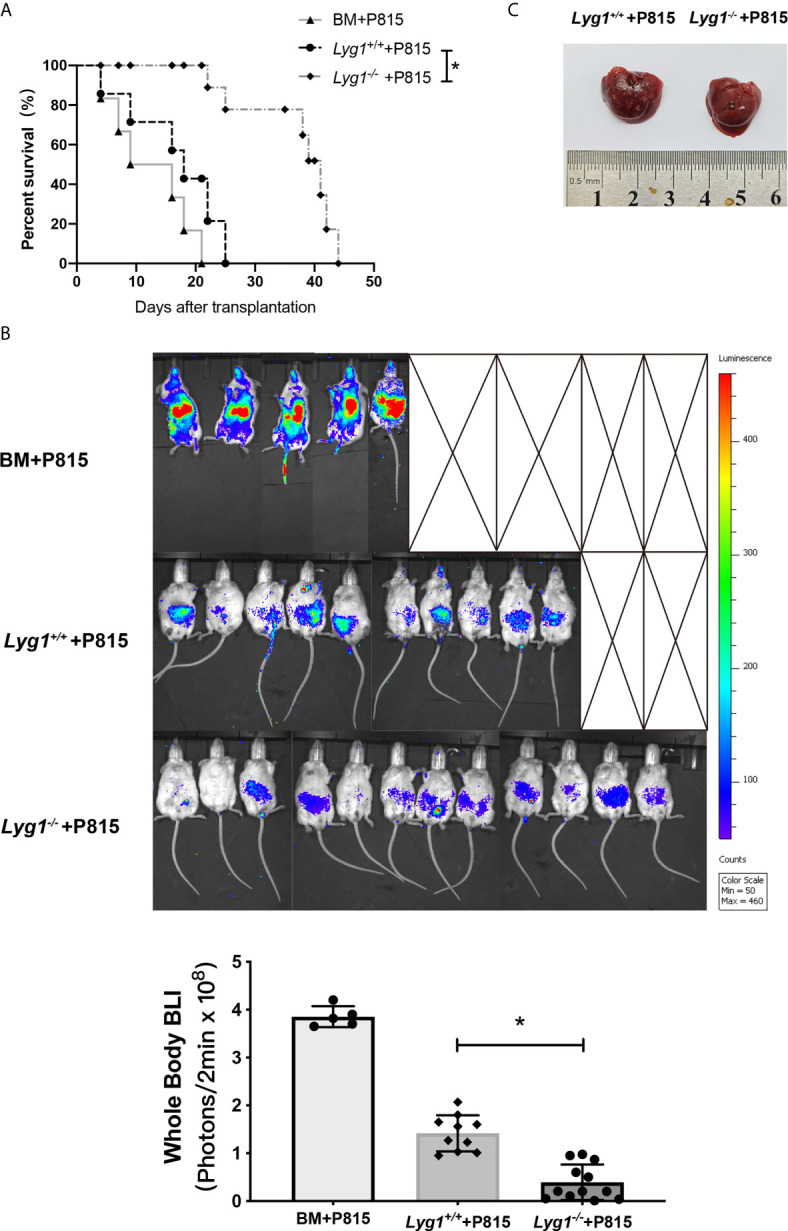
LYG1 deficiency in donor T cells preserved GVT response. Lethally irradiated BALB/c mice were reconstituted with 5 × 10^6^ B6 BM with or without 3 × 10^6^ T cells from *Lyg1^+/+^* or *Lyg1^-/-^* mice, followed by 2.5 × 10^4^ P815 cells (H2Kd) injected intravenously. **(A)** Survival after transplantation was monitored. **(B)** Tumor growth was monitored using bioluminescence imaging on day 14. Bioluminescence was quantified using whole body with Living Image software. Whole body images are shown and statistical results of average bioluminescence intensities are expressed as the mean ± SD, *p < 0.05 compared with *Lyg1^+/+^* group. **(C)** Livers were excised when the mice died or on day 14 after transplantation. n = 12 for *Lyg1^+/+^* or *Lyg1^-/-^* mice group, n = 10 for BM group. *p < 0.05 compared with *Lyg1^+/+^* group.

To determine whether the reduction of aGVHD lethality by LYG1 deficiency would affect GVT activity, mouse mastocytoma cell strain P815 (H2Kd) were injected intravenously on day 0 to generate murine GVT model. The mice receiving *Lyg1^-/-^* T cells exhibited a higher survival rate, lower tumor signal and lower tumor burden than that of the mice receiving *Lyg1^+/+^* T cells and BM cells (**Figures 8A–C**). Furthermore, there was no evident GVHD as the time of death in GVT model mice. The results suggested that targeting LYG1 might be an alternative to ameliorating aGVHD without impairing GVT function.

## Discussion

In this study, the role and mechanisms of LYG1 in aGVHD were explored. We demonstrated that mice receiving *Lyg1^-/-^* donor T cells alleviated aGVHD, increased long-term survival rates, showed less weight loss, lower GVHD clinical pathological scores and milder tissues damages, than mice receiving *Lyg1^+/+^* donor T cells in CD3^+^ or CD4^+^ T cells transplanting-major MHC mismatched aGVHD model and in haplo-HSCT model. Additionally, rhLYG1 intraperitoneally administration aggravated aGVHD severity, which confirmed the results established in the *Lyg1^-/-^* mice. Furthermore, we discovered that LYG1 deficiency in donor T cells can decrease infiltration of alloreactive CD4^+^ T cells in aGVHD mice target organs, inhibit alloreactive of CD4^+^ T cells and Th1 differentiation, promote Treg differentiation of allogeneic CD4^+^ T cells *in vitro* and *in vivo*.

Donor-derived CD4^+^ T cells are particularly important in the pathogenesis of aGVHD. A large number of clinical trials have taken CD4^+^ T cells as a potential target for GVHD treatment (3). Firstly we proved that LYG1 mediated GVHD development mainly through CD4^+^ T cells, but not CD8^+^ T cells by using purified CD4^+^ and CD8^+^ T cells as grafts. In aGVHD, alloreactive CD4^+^T cells are directed by chemokines and migrate to target tissues and organs where they cause tissue injury (20–22). CXCL9, CXCL10-CXCR3 interactions has been linked to activated T cell trafficking to aGVHD target organs in humans and mice (16). Our previous study found that rhLYG1 administration in mice can enhance the expression of T cell chemokines, including CCL5, CXCL9 and CXCL10, and infiltration of T cells in tumors (11). In this study, the decreased infiltration of allogeneic CD4^+^ T cells in the livers and lungs of mice that received *Lyg1^-/-^* T cells, which maybe related that LYG1 deficiency inhibited the expression of CXCL9 and CXCL10, explained partially that LYG1 deficiency in donor T cells suppressed aGVHD.

Another mechanism for LYG1 deficiency alleviating GVHD was able to inhibit IFN-γ production of donor derived T cells. IFN-γ plays an important promoting role in the alloreactivity of donor derived T cells in aGVHD (23). However, other studies have found that IFN-γ played a protective role against aGVHD, which depended on the time phase of IFN-γ production in allo-HSCT (23–25). Exogenous injection of IL-12 or IL-18 increased the expression of IFN-γ, thereby inducing the expression of Fas in donor T cells, leading to activation-induc,ed cell death, reducing donor T cells responses to host antigens and finally attenuating aGVHD (26, 27). Our previous studies have shown that rhLYG1 can promote antigen specific activity and IFN-γ production of T lymphocytes in tumor models (11). In this study, we found inhibited activation of CD4^+^ T cells and IFN-γ expression of allogeneic T cells in mice receiving *Lyg1^-/-^* donor T cells, whereas rhLYG1 administration aggravated aGVHD severity through promoting IFN-γ production of allogeneic T cells, more importantly, the absence of IFN-γ in donor T cells could partially abrogate rhLYG1-induced GVHD development, corroborating that the effect of LYG1 on aGVHD were mainly mediated by IFN-γ.

Treg cells play a significant role in maintaining tolerance in aGVHD by limiting T cell function (28). Many studies have proven that therapeutic modulation or adoptive transfer of Treg can directly prevent GVHD (29). CD8^+^Foxp3^+^ T cells, a Treg subpopulation, can be induced and ameliorate GVHD in mouse models (30). In our study, LYG1 deficiency led to the enhanced proportions of Treg cells *in vitro*. Similarly, the absence of LYG1 in donor T cells increased the proportions of allogeneic Treg (CD4^+^Foxp3^+^ T cells and CD8^+^Foxp3^+^ T cells) in different GVHD models *in vivo*. These results provided another explanation that LYG1 deficiency in donor T cells alleviated GVHD. Importantly, CD8^+^Foxp3^+^ Treg cells display cytotoxic activity which can suppress tumor during GVHD (31). These results explained partially if not fully that LYG1 deficiency in donor T cells suppressing GVHD while preserving GVT effect.

Our study demonstrated that LYG1 deficiency in donor T cells suppressed Th1 cells and promoted Treg cells differentiation in aGVHD model. Th cell differentiation is regulated by multiple cytokines and transcription factors. In the absence of IL-6, TGF-β stimulates a transcriptional program in naive CD4^+^ T cells with Foxp3 up-regulation and leads the evolvement of Treg cells (32).TNF-α blockade was shown to increase Foxp3 expression in patients with RA (33, 34). In this study, we found that the absence of LYG1 in donor T cells reduced the production of IL-6 and TNF-α in different GVHD models. Therefore, we speculated that LYG1 deficiency promoted Treg cells differentiation by inhibiting IL-6 and TNF-α. T-bet is a transcriptional activator of IFN-γ and orchestrates the cell-migratory program by directly controlling expression of the chemokine receptors CXCR3 (18). We showed that the absence of LYG1 decreased the expression of T-bet and CXCL10 in GVHD models. It was supposed that LYG1 deficiency suppressed Th1 cells polarization *via* inhibiting T-bet pathway related with CXCL10-CXCR3 axis, which was consistent with this report (35).

As a secretory protein, the cell sources of LYG1 are unclear. In this study, we demonstrated the role of LYG1 in aGVHD using WT BM and *Lyg1^+/+^* or *Lyg1^-/-^* T cells as the graft, but not KO BM and *Lyg1^+/+^* or *Lyg1^-/-^* T cells as the graft. Because only transplantation of allogeneic BM did not induce aGVHD, we excluded the effects of LYG1 derived from of BM in aGVHD mouse model we used in this study. Second, the recipients and its irradiation conditions of WT and KO groups were all the same, so we excluded the impacts of LYG1 derived from recipients in aGVHD mouse model we used. More importantly, rhLYG1 aggravated the aGVHD severity by promoting IFN-γ production and inhibiting Foxp3 expression, providing orthogonal validation for the results established using the *Lyg1^-/-^* mice.

In summary, we demonstrate LYG1 regulates aGVHD *via* altering the alloreactivity of CD4^+^ T cells and the balance of Th1 and Treg differentiation of allogeneic CD4^+^ T cells. Our study indicates that LYG1 may be a novel target in aGVHD by mitigating aGVHD without impairing GVT effect. The therapeutic effect of targeting LYG1 is required in future investigations.

## Data Availability Statement

The original contributions presented in the study are included in the article/**Supplementary Material**. Further inquiries can be directed to the corresponding authors.

## Ethics Statement

The animal study was reviewed and approved by Ethics Committee of Peking University First Hospital. Written informed consent was obtained from the owners for the participation of their animals in this study.

## Author Contributions

HL contributed conception and design of the study, performed the most experiments of this study and wrote the paper. ZY performed the MLR assay and part of aGVHD models. BT, SM, and CQ helped to establish aGVHD mouse models. YL, ZL, YS, and YZ performed part of flow cytometric analysis. QW, MY, and ZS performed part of qPCR experiments. HR and YD contributed conception and design of the study. All authors contributed to the article and approved the submitted version.

## Funding

This study was supported by grant from The National Natural Science Foundation of China (NSFC) (Grant Number 81600144, 82071757 and 81970160) and grant from Beijing Natural Science Foundation (Grant Number 7212115).

## Conflict of Interest

The authors declare that the research was conducted in the absence of any commercial or financial relationships that could be construed as a potential conflict of interest.

## Supplementary Material

Supplementary Figure 1 Gating strategies for flow cytometry analyses presented in **Figure 1**. **(A–C)** The expression of CD69 **(A)**, IFN-γ **(B)** and Foxp3 **(C)** in isotype (ISO) (left) and negative control without stimulating cells (right).

Supplementary Figure 2 Gating strategies for flow cytometry analyses in T cells before adoptive transfer presented in **Figure 2** **(A)**. **(B, C)** Representative flow cytometry plots and frequencies of naive (CD44^lo^CD62L^hi^), central memory (CD44^hi^CD62L^hi^), effector (CD44^hi^CD62L^lo^) in CD4^+^ T cells and CD8^+^ T cells respectively. n=5 per group. **(D, E)** The percentages of CD3^+^ T, CD4^+^ T and CD8^+^ T cells in BM cells from donor mice before adoptive transfer.

Supplementary Figure 3 LYG1 deficiency reduced allogeneic T cells function in haploidentical transplant model. Splenocytes of recipient mice were isolated on day 14 after transplantation and analyzed by flow cytometry. **(A, B)** The percentages of CD69 expression in CD4^+^ T cells and CD8^+^ T cells. **(C, D)** The percentages of IFN-γ expression in CD4^+^ T cells and CD8^+^ T cells. **(E, F)** The percentages of Foxp3 expression in CD4^+^ T cells and CD8^+^ T cells. n=5 per group.

Supplementary Figure 4 Gating strategies for flow cytometry analyses presented in **Figure 3**. The expression of CD69 **(A, B)**, CD44 and CD62L **(C, D)**, IFN-γ **(E, F)** and Foxp3 **(G, H)** in CD4^+^ T cells and CD8^+^ T cells respectively in ISO (left), BM control (middle) and Syn control (right).

Supplementary Figure 5 The concentrations of IFN-γ, TNF-α and IL-6 in serum in aGVHD models. **(A)**The concentrations of IFN-γ, TNF-α and IL-6 in serum at day 7 after transplantation in major MHC mismatched aGVHD model. **(B)**The concentrations of IFN-γ, TNF-α and IL-6 in serum at day 14 after transplantation in haploidentical model. **(C, D)** The concentrations of IFN-γ, TNF-α and IL-6 in serum at day 7 after transplantation in purified CD4^+^ T cells or CD8^+^ T cells. n=4-5 per group. Statistical results are expressed as the mean ± SD, *p< 0.05 compared with *Lyg1^+/+^* group.

## Abbreviations

aGVHD, acute graft-*versus*-host disease; allo-HSCT, allogeneic allogeneic hematopoietic cell transplantation; BM, Bone marrow cells; Treg, Regulatory T cells; Th1, T helper 1 cells; Tc1, T cytotoxic 1 cells; rhLYG1, recombinant human LYG1 protein; LYG1, Lysozyme G-like 1; MLR, mixed lymphocyte reaction; TBI, total body irradiation; GVT, graft-*versus*-tumor.
