# Corrigendum: LYG1 Deficiency Attenuates the Severity of Acute Graft-Versus-Host Disease *via* Skewing Allogeneic T Cells Polarization Towards Treg Cells

**DOI:** 10.3389/fimmu.2021.762728

**Published:** 2021-09-16

**Authors:** Huihui Liu, Zhengyu Yu, Bo Tang, Shengchao Miao, Chenchen Qin, Yuan Li, Zeyin Liang, Yongjin Shi, Yang Zhang, Qingya Wang, Miao Yan, Zhengyang Song, Hanyun Ren, Yujun Dong

**Affiliations:** Department of Hematology, Peking University First Hospital, Beijing, China

**Keywords:** LYG1, aGVHD, allogeneic CD4+ T cells, alloreactivity, Th1 cells, Treg cells

In the original article, there was a mistake in **Figure 3F** as published. **Figure 3F** contains a duplicated FACS plot in Lyg1-/- group. We used the graph of Lyg1-/- group as that of Lyg1+/+ group by mistake. The corrected **Figure 3** appears below.

**Figure 3:**
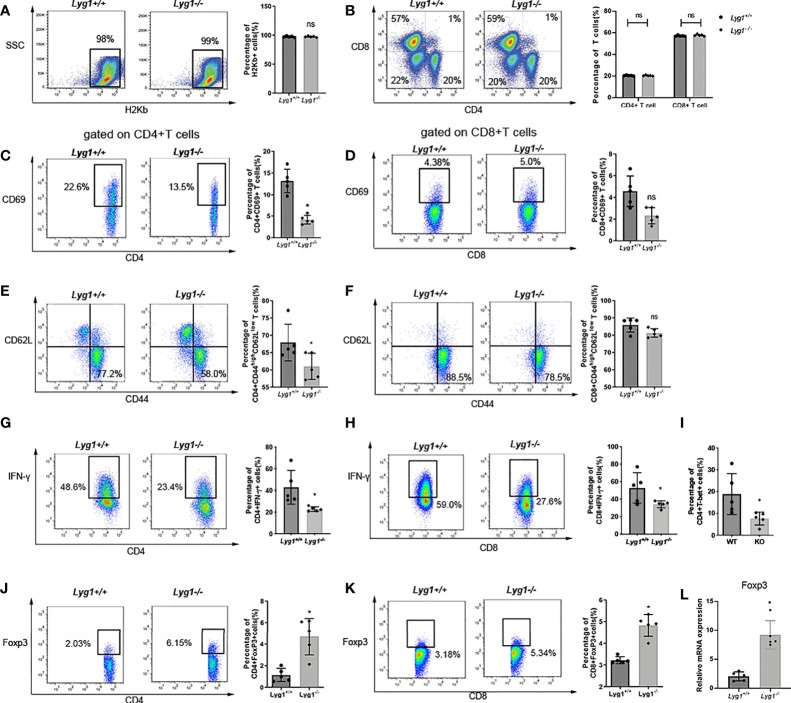
LYG1 deficiency reduced allogeneic T cells function in spleens. Splenocytes of recipient mice were isolated on day 7 after transplantation and analyzed by flow cytometry and qPCR. **(A)** The percentages of H2Kb+ cells in living splenocytes. **(B)** The percentages of CD4+ T and CD8+ T cells in H2Kb+ splenocytes. **(C, D)** The percentages of CD69 expression in CD4+ T cells and CD8+ T cells. **(E, F)** The expression of effector (CD44hiCD62Llo) phenotype gated on CD4+ T and CD8+ T cells. **(G, H)** The percentages of IFN-g expression in CD4+ T cells and CD8+ T cells. **(I)** The percentages of T-bet expression in CD4+ T cells. **(J, K)** The percentages of Treg in CD4+ T cells and CD8+ T cells. The percentages of Figure 3 **(C–K)** were all gated on H2Kb+CD4+ cells or H2Kb+CD8+ cells. **(L)** Foxp3 expression of splenocytes were examined by qPCR. Independent experiment was performed 3 times. The results in the repeats were similar. n = 5 per group. Representative plots are shown and statistical results are expressed as the mean ± SD, *p < 0.05 compared with Lyg1+/+ group. ns, no significance.

The authors apologize for this error and state that this does not change the scientific conclusions of the article in any way. The original article has been updated.

## Publisher’s Note

All claims expressed in this article are solely those of the authors and do not necessarily represent those of their affiliated organizations, or those of the publisher, the editors and the reviewers. Any product that may be evaluated in this article, or claim that may be made by its manufacturer, is not guaranteed or endorsed by the publisher.

