# Supplementary figures and images for: LYG1 Deficiency Attenuates the Severity of Acute Graft-Versus-Host Disease via Skewing Allogeneic T Cells Polarization Towards Treg Cells
Source: Front Immunol. 2021 Jun 28;12:647894. doi: 10.3389/fimmu.2021.647894 (PMC8273552; doi:10.3389/fimmu.2021.647894)

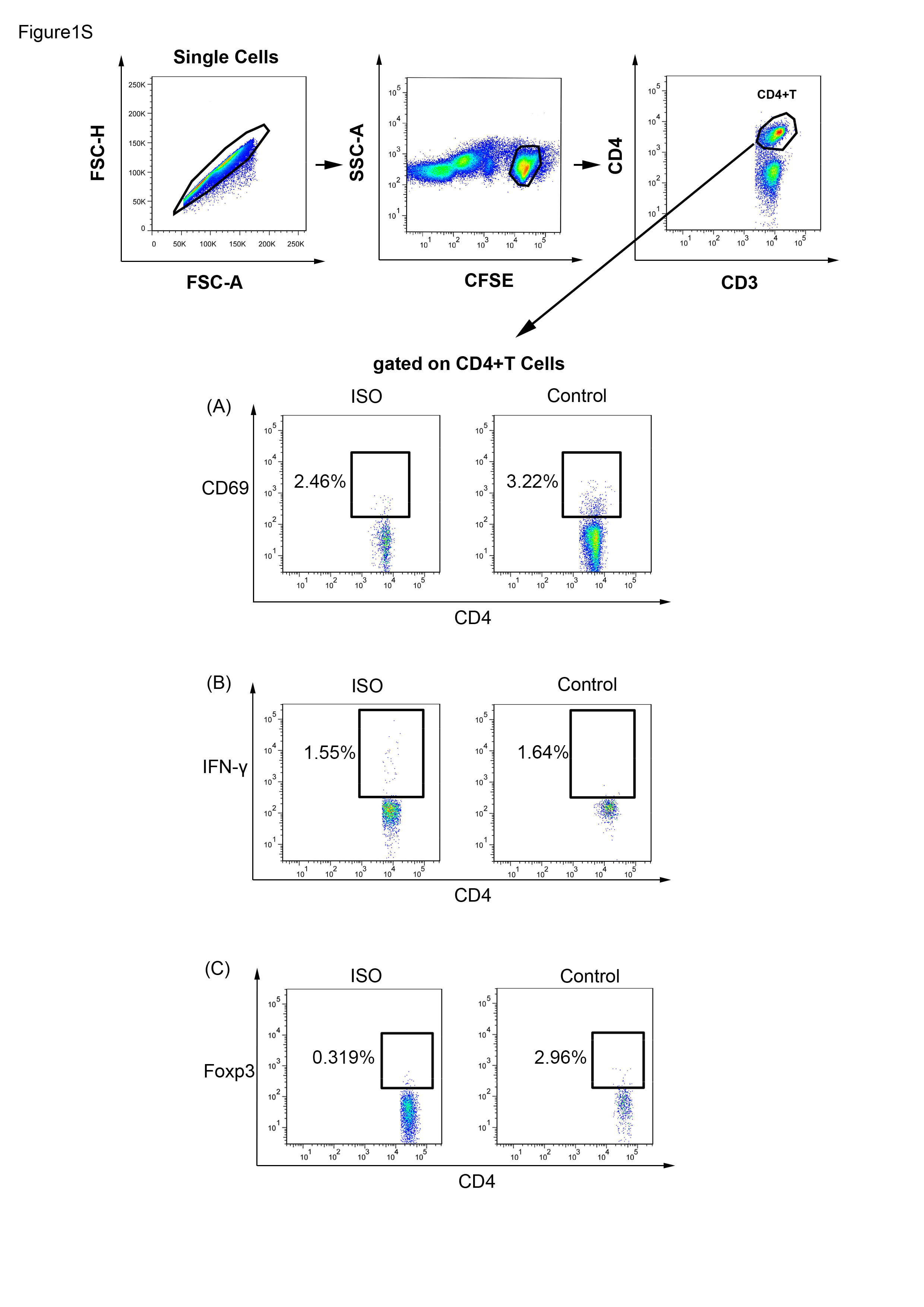

Supplement: Supplementary Figure 1 — Gating strategies for flow cytometry analyses presented in Figure 1. (A–C) The expression of CD69 (A), IFN-γ (B) and Foxp3 (C) in isotype (ISO) (left) and negative control without stimulating cells (right). [file Image_1.tif]

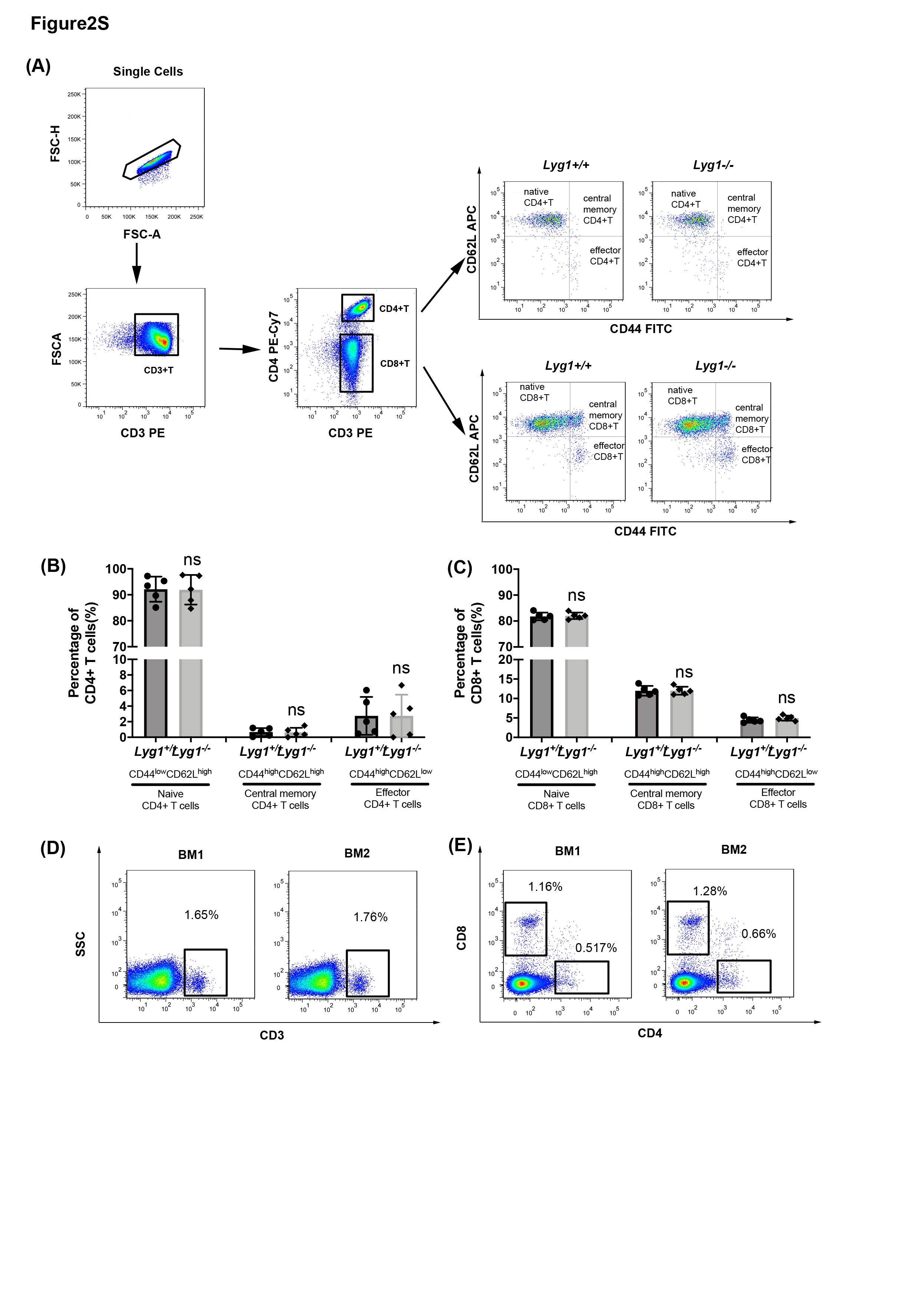

Supplement: Supplementary Figure 2 — Gating strategies for flow cytometry analyses in T cells before adoptive transfer presented in Figure 2 (A). (B, C) Representative flow cytometry plots and frequencies of naive (CD44loCD62Lhi), central memory (CD44hiCD62Lhi), effector (CD44hiCD62Llo) in CD4+ T cells and CD8+ T cells respectively. n=5 per group. (D, E) The percentages of CD3+ T, CD4+ T and CD8+ T cells in BM cells from donor mice before adoptive transfer. [file Image_2.tif]

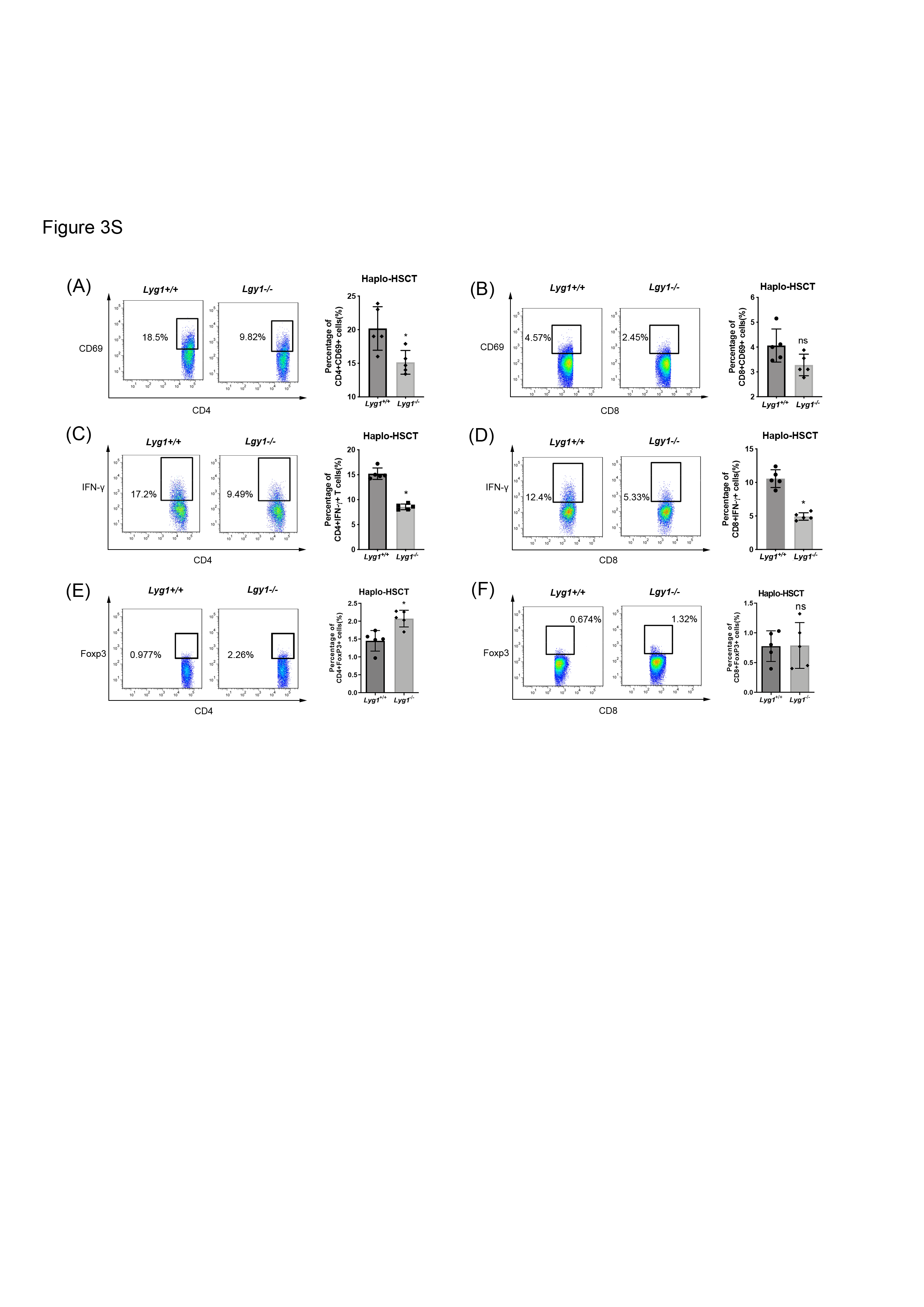

Supplement: Supplementary Figure 3 — LYG1 deficiency reduced allogeneic T cells function in haploidentical transplant model. Splenocytes of recipient mice were isolated on day 14 after transplantation and analyzed by flow cytometry. (A, B) The percentages of CD69 expression in CD4+ T cells and CD8+ T cells. (C, D) The percentages of IFN-γ expression in CD4+ T cells and CD8+ T cells. (E, F) The percentages of Foxp3 expression in CD4+ T cells and CD8+ T cells. n=5 per group. [file Image_3.tif]

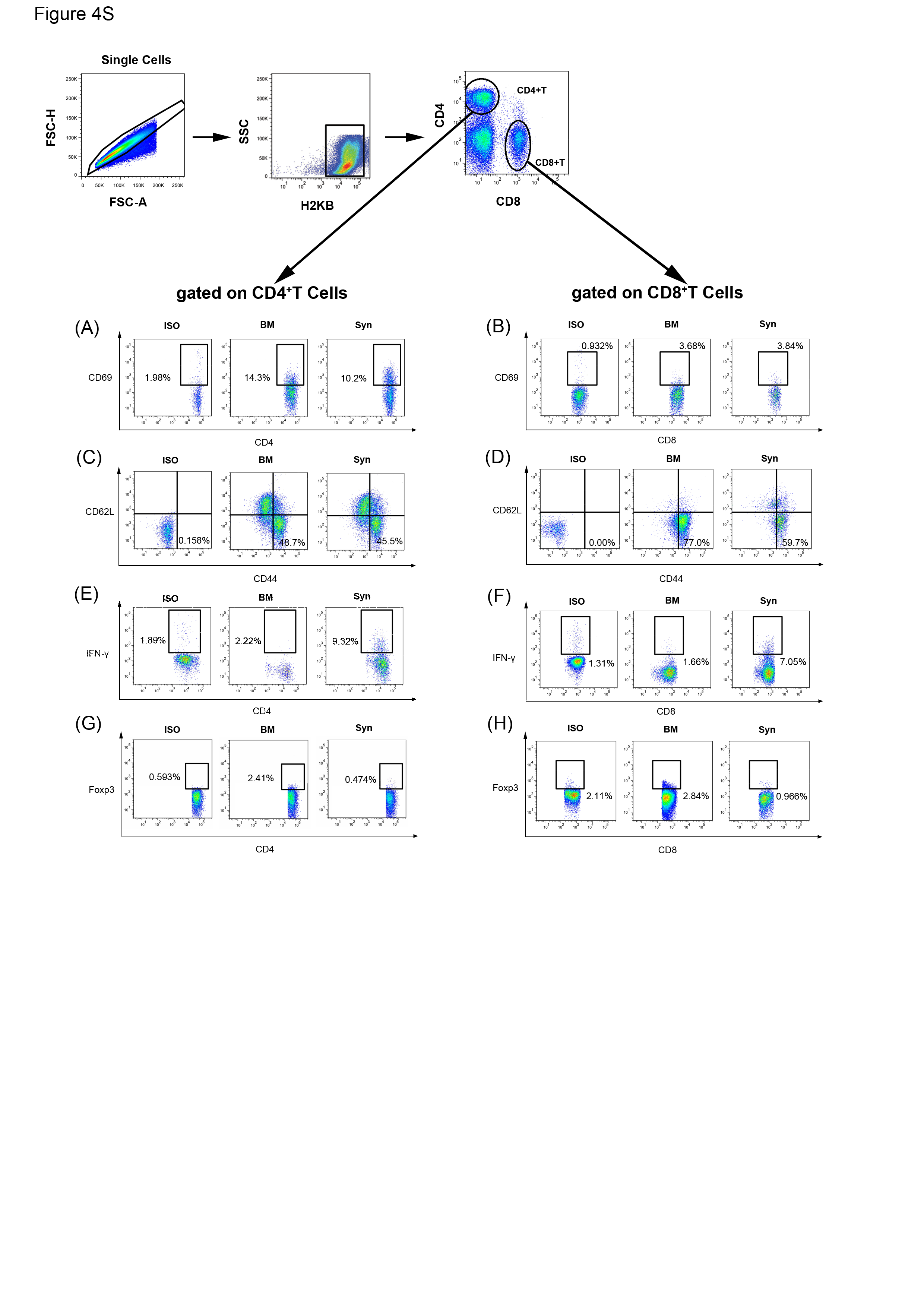

Supplement: Supplementary Figure 4 — Gating strategies for flow cytometry analyses presented in Figure 3. The expression of CD69 (A, B), CD44 and CD62L (C, D), IFN-γ (E, F) and Foxp3 (G, H) in CD4+ T cells and CD8+ T cells respectively in ISO (left), BM control (middle) and Syn control (right). [file Image_4.tif]

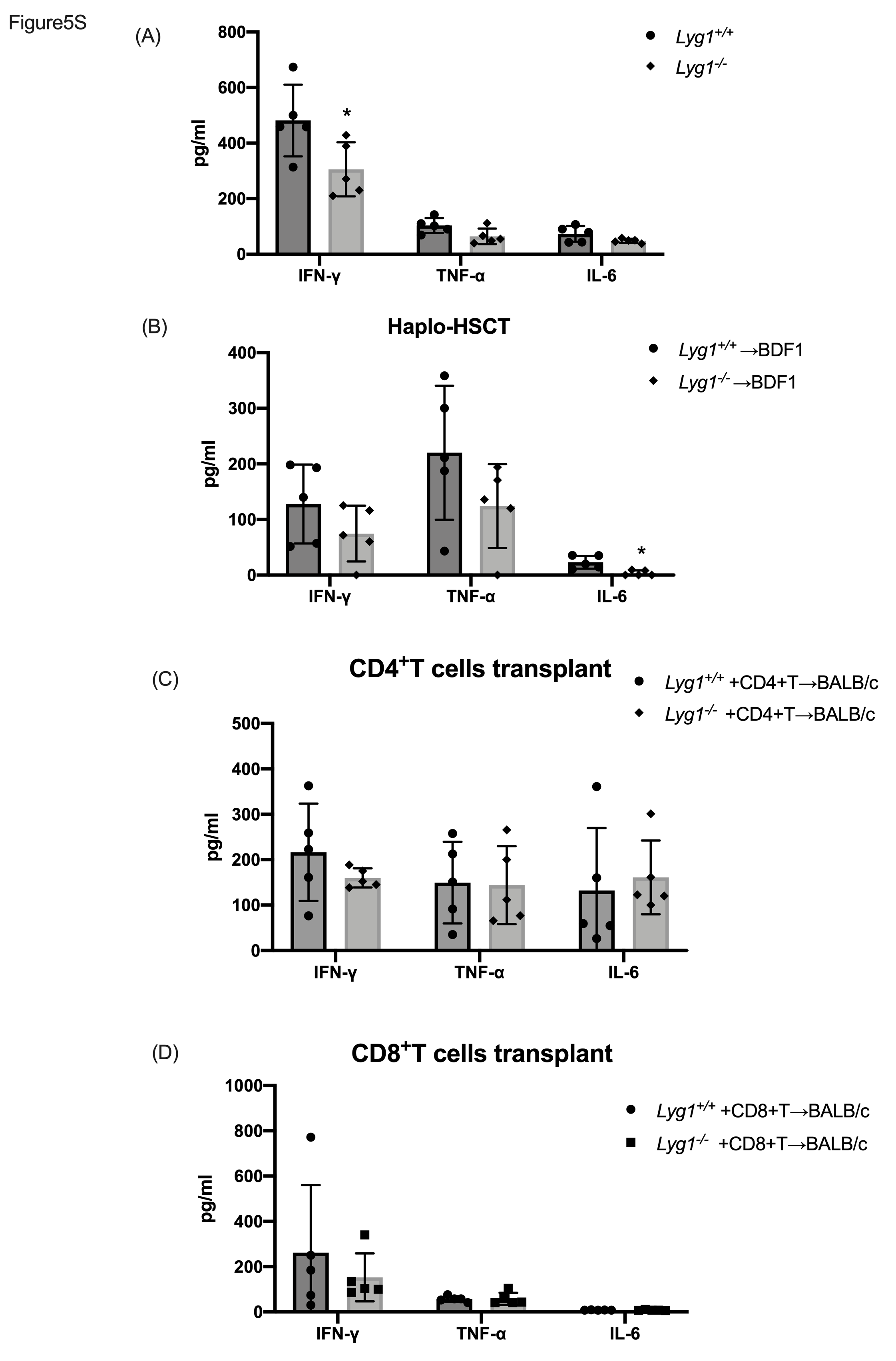

Supplement: Supplementary Figure 5 — The concentrations of IFN-γ, TNF-α and IL-6 in serum in aGVHD models. (A)The concentrations of IFN-γ, TNF-α and IL-6 in serum at day 7 after transplantation in major MHC mismatched aGVHD model. (B)The concentrations of IFN-γ, TNF-α and IL-6 in serum at day 14 after transplantation in haploidentical model. (C, D) The concentrations of IFN-γ, TNF-α and IL-6 in serum at day 7 after transplantation in purified CD4+ T cells or CD8+ T cells. n=4-5 per group. Statistical results are expressed as the mean ± SD, *p< 0.05 compared with Lyg1+/+ group. [file Image_5.tiff]
